# Mathematical Modeling of the Gastrointestinal System for Preliminary Drug Absorption Assessment

**DOI:** 10.3390/bioengineering11080813

**Published:** 2024-08-09

**Authors:** Antonio D’Ambrosio, Fatjon Itaj, Filippo Cacace, Vincenzo Piemonte

**Affiliations:** 1 Unit of Chemical-Physics Fundamentals in Chemical Engineering, Department of Science and Technology for Sustainable Development and One Health, University Campus Bio-Medico of Rome, Via Alvaro del Portillo 21, 00128 Rome, Italy; fatjon.itaj@alcampus.it (F.I.); v.piemonte@unicampus.it (V.P.); 2 Research Unit of Computer Systems and Bioinformatics, Department of Engineering, University Campus Bio-Medico of Rome, Via Alvaro del Portillo 21, 00128 Rome, Italy; f.cacace@ieee.org

**Keywords:** pharmacokinetic, drug absorption, gastrointestinal system, PBPK model, in silico simulation

## Abstract

The objective of this study is to demonstrate the potential of a multicompartmental mathematical model to simulate the activity of the gastrointestinal system after the intake of drugs, with a limited number of parameters. The gastrointestinal system is divided into five compartments, modeled as both continuous systems with discrete events (stomach and duodenum) and systems with delay (jejunum, ileum, and colon). The dissolution of the drug tablet occurs in the stomach and is described through the Noyes–Whitney equation, with pH dependence expressed through the Henderson–Hasselbach relationship. The boluses resulting from duodenal activity enter the jejunum, ileum, and colon compartments, where drug absorption takes place as blood flows countercurrent. The model includes only three parameters with assigned physiological meanings. It was tested and validated using data from in vivo experiments. Specifically, the model was tested with the concentration profiles of nine different drugs and validated using data from two drugs with varying initial concentrations. Overall, the outputs of the model are in good agreement with experimental data, particularly with regard to the time of peak concentration. The primary sources of discrepancy were identified in the concentration decay. The model’s main strength is its relatively low computational cost, making it a potentially excellent tool for in silico assessment and prediction of drug adsorption in the intestine.

## 1. Introduction

The absorption of drugs through the gastrointestinal (GI) tract is a crucial factor in determining their therapeutic efficacy and safety profile [1]. For this reason, in pharmacology and drug development, there is an ongoing effort to understand the mechanisms that govern this phenomenon [2]. On the whole, GI drug absorption is a complex process influenced by various factors, such as physiological differences across different regions of the GI tract, drug physicochemical properties, and interindividual variability [3,4,5]. The intricate interplay between physiological processes and drug behavior makes it challenging to create a comprehensive model, without unrealistic and oversimplified assumptions.

In recent years, the use of physiologically based pharmacokinetic (PBPK) models has revolutionized the ability to predict and comprehend drug absorption dynamics with unparalleled precision [6,7]. PBPK models can be used to describe or predict the pharmacokinetics of a drug in specific individuals or under certain physiological or pathological conditions. Their primary outcome is a set of concentration–time curves that illustrate the temporal behavior of the drug in blood, plasma, and/or other relevant organs [8].

The increasing physiological and anatomical knowledge, along with the development of this scientific branch, has led to the creation of various modeling methodologies [9,10,11], which can guide drug research and development, predicting pharmacological interactions [12,13]. In all cases, PBPK models simulate the structure of a living organism by representing its various organs and tissues as compartments, connected through a circulatory blood circuit [8]. The latter can be further divided into arterial and venous pools, if necessary, for the pharmacokinetics of the drug and the objective [14]. The selection of compartments generally relies on the model’s objective and the physicochemical and pharmacological properties of the drug being modeled [15]. Tissues with similar physiological, physicochemical, and biochemical properties are grouped into a single compartment. On the other hand, tissues with distinct properties, such as the liver, where metabolism occurs, or target tissues, are isolated as lumped compartments [16,17].

Physiological parameters, such as organ/tissue volumes, cardiac output, blood flows, tissue composition, surface area, pH values, and gastrointestinal transit times, are essential for incorporating into PBPK models [18,19]. These parameters characterize the anatomical structure and physiological processes of the modeled species and are known to vary among species, individuals, ages, and physiological/pathological states [8,20]. Other useful pieces of information that can be integrated into the model equations include the effects of food [21], aging [22,23], rest and physical exertion [24,25], and gender differences [26].

PBPK modeling was initially developed for the pharmaceutical industry and has since been applied in pharmacology, drug development, drug discovery, and preclinical support [13,27,28]. When used in conjunction with in vitro data and physicochemical characteristics, it can predict the pharmacokinetics of potential drug candidates in animals, resulting in a reduction in unnecessary animal testing and significant time savings [29]. A PBPK model can also assist in choosing one compound over another and can provide more confidence in dosing decisions, reducing the time and costs of clinical trials [13]. Furthermore, it can provide information on pharmacokinetics in various physiological and pathological conditions, even during surgery [30,31,32]. To enhance safety and improve the success rate of drug development, it is recommended to estimate the pharmacokinetics of both the main drug and its metabolite [33,34]. This can help predict drug interactions and provide an overview of the effects of concurrent drug administration [35].

Focusing on GI drug adsorption, different multicompartmental PBPK models have been proposed in the literature [4,36,37,38]. Particularly, there are several advanced models that consider multiple biological and biochemical factors to investigate the intricate mechanisms underlying drug absorption in the gut [39,40]. While they provide insightful predictions of the variability of drug availability in populations, this is often achieved at a high computational cost [41,42]. Furthermore, an elevated number of parameters is often required, which is difficult to support by adequate experimental campaigns [41]. In light of these considerations, a simplified model, even if less sophisticated, can be a valuable tool during the early stages of drug testing, when limited experimental data are available. In fact, it may offer a rapid preliminary estimation of parameters, which can subsequently be integrated into more detailed models. For example, Pompa et al. [43] developed a multicompartmental model based on the principles of chemical engineering. It describes compartments where the major absorption occurs as plug-flow reactors and incorporates a dependence on ingested food in the digestive process. Cacace et al. [44] partially corrected the model proposed by Pompa et al. from a physiological perspective by including the actual behavior during fasting, assuming the discrete movement of boluses, driven by peristaltic waves.

In this context, this study presents a multicompartmental mathematical model that describes drug concentrations in the bloodstream. The model extends the work of Cacace et al. by including the ileum and colon and incorporating the digestive process, to represent experimental data with increasing accuracy. The objective is to demonstrate the potential of a model defined by a reduced number of parameters in the preliminary evaluation of drug bioavailability. Consequently, a series of assumptions were formulated with the intention of optimizing computational cost, as will be further elucidated.

## 2. Materials and Methods

**Figure 1:**
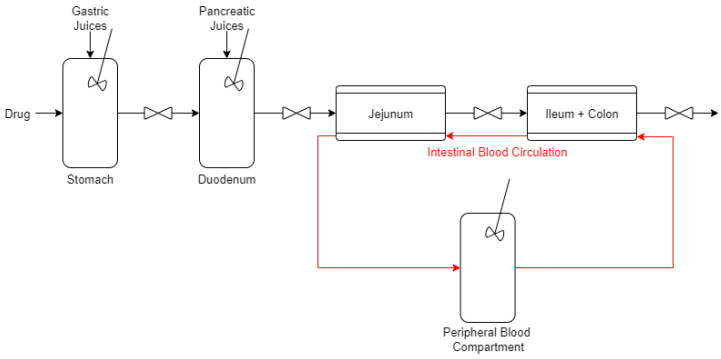
Model of the gastrointestinal system, with the five compartments considered.

As illustrated in Figure 1, the gastrointestinal system is described through a four-compartment model, including the stomach, duodenum, jejunum, and ileum–colon. Based on the literature [45,46], drug absorption primarily occurs in the jejunum and the ileum, whereas the colon does not play a key role, unless the drug is engineered for adsorption in this intestinal segment. For this reason, the colon was not modeled separately, but was incorporated into the ileum compartment. Additionally, in light of the fact that in clinical studies blood is typically drawn from the right or left arm to detect drug concentration, it was deemed necessary to integrate the peripheral circulation compartment into the model, which is connected to the jejunum and the ileum–colon.

The maximum duration of the simulation is dependent on the compartment. It was assumed that the maximum simulation time would be equal for the stomach and duodenum compartments, on the basis of the initial volume (i.e., the amount of the meal). Consequently, in the event that the volume falls below a minimum threshold, the simulation is terminated. Conversely, the maximum simulation time for the jejunum and ileum–colon compartments depends on the maximum time of concentration detection in scientific studies from which the data were collected.

Perfect drug dissolution is assumed, which implies complete dissolution in the stomach. This means that the drug arrives in the duodenum completely dissolved. This assumption is considered reasonable given the tolerance of the pyloric sphincter for the passage of solids up to 2 mm [47].

### 2.1. Stomach and Duodenum

The stomach and duodenum are modeled as discrete event continuous systems (DESs) [48]. A set of balance equations for volume and drug was formulated for both compartments [43]:

(1) $$\frac{dV_{s}\left(t\right)}{dt}=Q_{s,in}\left(t\right)-Q_{s,out}\left(t\right)+Q_{gj}$$

(2) $$\frac{d}{dt}\left(V_{s}\left(t\right)\cdot C_{s}\left(t\right)\right)=Q_{s,in}\left(t\right)C_{0}-Q_{s,out}\left(t\right)C_{s}\left(t\right)+k_{s}^{d}\left(S_{s}-C_{s}\left(t\right)\right)$$

(3) $$\frac{dV_{d}\left(t\right)}{dt}=Q_{d,in}\left(t\right)-Q_{d,out}\left(t\right)+Q_{pj}$$

(4) $$\frac{d}{dt}\left(V_{d}\left(t\right)\cdot C_{d}\left(t\right)\right)=Q_{d,in}\left(t\right)C_{s}\left(t\right)-Q_{d,out}\left(t\right)C_{d}\left(t\right)$$

In Equations (1)–(4), $V_{i}$ and $C_{i}$ are the volume and the concentration of drug in compartment *i* (*s* = stomach and *d* = duodenum), $Q_{i,in}$ and $Q_{i,out}$ are the inlet and outlet flow rates, and $Q_{gj}$ and $Q_{pj}$ are the flow rates of gastric and pancreatic juices, respectively. In addition, $C_{0}$ represents the initial drug concentration in the meal, whereas the term $k_{s}^{d}\left(S_{s}-C_{S}\right)$ accounts for the dissolution of the drug tablet, which occurs exclusively in the stomach. This dissolution process is described by the Noyes–Whitney equation [49,50] (Equations (5) and (6)), with a pH dependency expressed by the Henderson–Hasselbach equation [51] (Equation (7)):

(5) $$\frac{dr_{p}\left(t\right)}{dt}=-4\pi \rho r_{p}^{2}k_{s}^{d}\left(S_{s}-C_{s}\left(t\right)\right)$$

(6) $$k_{s}^{d}=\frac{3DS_{s}4\pi }{\rho }$$

(7) $$S_{s}=S_{0}\left(1+10^{pH-pk_{a}}\right)$$

$S_{0}$ is the water solubility, ρ is the drug density, *D* is the diffusion coefficient in the stomach, and $r_{p}$ is tablet radius. The parameters assumed for each drug are listed in Table 1.

As this model is intended for preliminary assessment, only major drug absorption sites are included. Therefore, Equations (1)–(4) do not include terms related to drug absorption, which mostly occurs in the jejunum and ileum, due to their extensive surface area and prolonged transit time [54,55].

Each discrete event is associated with the drainage of chyme from the stomach into the duodenum and the corresponding emptying of the duodenum into the jejunum. Gastric and pancreatic juice production, being an endogenous process, is continuous over time. The simulation begins upon ingestion of the meal and subsequent administration of the drug pill, both of which are already present in the stomach. Consequently, the $Q_{s,in}=0$, and the initial volume and concentration of the drug in the stomach depend on the composition of the meal, based on the experimental data. The latter were derived from clinical trials that were previously documented in the medical literature and are detailed in Table 2.

In order to account for discrete events, it is necessary to provide the system of Equations (1)–(4) with appropriate continuity conditions, described in Equations (8)–(12):

(8) $$Q_{s,out}\left(t_{k}^{+}\right)=Q_{d,in}\left(t_{k}^{+}\right)=\frac{V_{s}\left(t_{k}^{-}\right)}{20}\cdot \frac{1}{1\;s}$$

(9) $$V_{s}\left(t_{k}^{+}\right)=\frac{19}{20}\cdot V_{s}\left(t_{k}^{-}\right)$$

(10) $$C_{s}\left(t_{k}^{+}\right)=C_{s}\left(t_{k}^{-}\right)=C_{d}\left(t_{k}^{+}\right)$$

(11) $$Q_{d,out}\left(t_{k}^{-}\right)=\frac{V_{d}\left(t_{k}^{-}\right)}{1\;s}$$

(12) $$V_{d}\left(t_{k}^{+}\right)=\frac{1}{20}\cdot V_{s}\left(t_{k}^{-}\right)$$

where $t_{k}$ represents the time at which the discrete event occurs, whereas the notation $f(t_{k}^{+})$ denotes $\lim_{t\to t_{k}^{+}}f\left(t\right)$ for each variable *f*. Similarly, $f\left(t_{k}^{-}\right)=\lim_{t\to t_{k}^{-}}f\left(t\right)$. It was also assumed that the gastric emptying time is 1 s, with an emptying frequency of 1.6 · $10^{-3}$ Hz. Therefore, every 600 s, the stomach discharges, in one second, 1/20 of its current volume into the duodenum. This parameter choice is based on the average time taken by the gastrointestinal system for digestion [47]. Although stomach emptying is not a discrete process and occurs continuously to a very small extent, given the nature of the model, the approximation is reasonable and functional.

It should be noted that the duodenum does not receive chyme from the stomach until the completion of the entire digestive process, which is only after the duodenum is completely emptied into the jejunum. The dynamics of duodenal activity are identical to those of the stomach, resulting in a complete emptying every 600 s. Subsequent filling occurs as chyme is received from the stomach. The initial conditions in the duodenum are dependent on the activity of the stomach, such that the value of the flow and the initial conditions following each emptying are equal to those leaving the stomach. The outputs of the duodenum are boluses that serve as inputs for the remainder of the intestine, with varying concentrations and volumes, resulting in distinct interactions within the intestine.

### 2.2. Jejunum and Ileum–Colon

**Figure 2:**
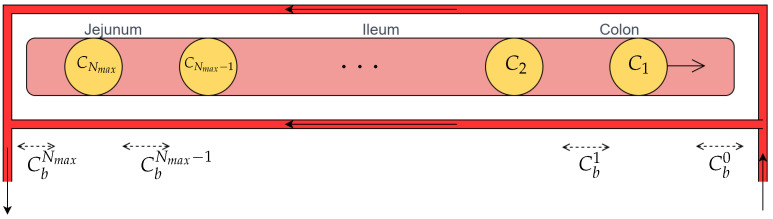
Schematic representation of boluses flowing in the intestine and exchanging drug with splanchnic circulation. $C_{b}^{i}$ refers to the drug concentration in the blood, while $C_{i}$ represents the concentrations of the drug in the boluses.

The jejunum and ileum–colon were modeled as cylindrical conduits, with boluses arriving as input, in accordance with the methodology described by Cacace et al. [44]. Each duodenal emptying event is associated with the release of a bolus, which traverses the intestine and exchanges drug with the blood, as depicted in Figure 2.

The peripheral blood compartment was, instead, modeled as a continuous stirred tank reactor (CSTR), in which a first-order reaction of drug degradation and consumption occurs. The splanchnic circulation was treated separately, with a countercurrent flow crossing both the ileum–colon and jejunum. Moreover, it was assumed that there was no segregation of the gut blood flow. As a result, Equations (13)–(15) were obtained:

(13) $$\frac{dC_{i}\left(t\right)}{dt}=-\alpha_{i}\left(C_{i}\left(t\right)-C_{b}^{i-1}\left(t\right)\right)$$

(14) $$C_{b}^{i}\left(t\right)-C_{b}^{i-1}\left(t\right)=K_{i}\left(C_{i}\left(t\right)-C_{b}^{i-1}\left(t\right)\right)$$

(15) $$V_{b}\frac{d{\bar{C}}_{b}}{dt}=Q_{b}\left(C_{b}^{N_{max}}-{\bar{C}}_{b}\right)-k_{d}{\bar{C}}_{b}V_{b}$$

where $C_{i}\left(t\right)$, $C_{b}^{i}\left(t\right)$, and ${\bar{C}}_{b}\left(t\right)$ are the concentrations of drug in the *i*-th bolus, in the blood in the intestine after the contact with the *i*-th bolus, and in the peripheral circulation, respectively. $C_{b}^{0}\left(t\right)$ is the concentration of drug in the blood at the entrance of the intestine and is linked to ${\bar{C}}_{b}\left(t\right)$ by Equation (16):

(16) $$C_{b}^{0}\left(t\right)={\bar{C}}_{b}\left(t-\delta \right)$$

where δ is the time that the blood remains in the peripheral circulation. $\alpha_{i}$ and $K_{i}$ are two parameters that are characteristic of the model [44], further defined in Section 2.3. They refer to the ileum–colon when $i=1,\cdots ,N_{max_{ic}}$ and to the jejunum when $i=N_{max_{ic}}+1,\cdots ,N_{max}$. $N_{max_{ic}}$ and $N_{max}$ are the maximum numbers of boluses transiting through the ileum–colon and the whole intestine, respectively (Equations (17) and (18)):

(17) $$N_{max_{ic}}=\left\lfloor \frac{\left(L_{i}+L_{c}\right)\cdot f_{e}}{u}\right\rfloor$$

(18) $$N_{max}=\left\lfloor \frac{\left(L_{j}+L_{i}+L_{c}\right)\cdot f_{e}}{u}\right\rfloor$$

The specific meanings and values of each parameter are delineated in Table 3.

### 2.3. Parameters Definition and Estimation

As previously described, the presented model results from the combination of two distinct models that were previously identified in the literature [43,44]. The primary novelty introduced lies in the definition of parameters: $\alpha_{j}$ for the jejunum, $\alpha_{ic}$ for the ileum–colon, and $k_{d}$, which represents the kinetic constant of drug degradation/consumption in the peripheral blood compartment. $\alpha_{j}$ and $\alpha_{ic}$ account for drug permeability and diffusivity through the intestinal barrier. Considering that drug absorption predominantly occurs in the jejunum and ileum, their values cannot be constant along the three intestinal segments. Therefore, it was assumed that both $\alpha_{j}$ and $\alpha_{ic}$ vary along the axial coordinate *x*, according to Cauchy distribution (Equation (19)):

(19) $$\alpha_{l}\left(x\right)=\frac{1}{\pi }\frac{\lambda_{l}}{1+{\left(x-{\tilde{L}}_{l}\right)}^{2}}\;\;\forall \;l=j,ic$$

**Figure 3:**
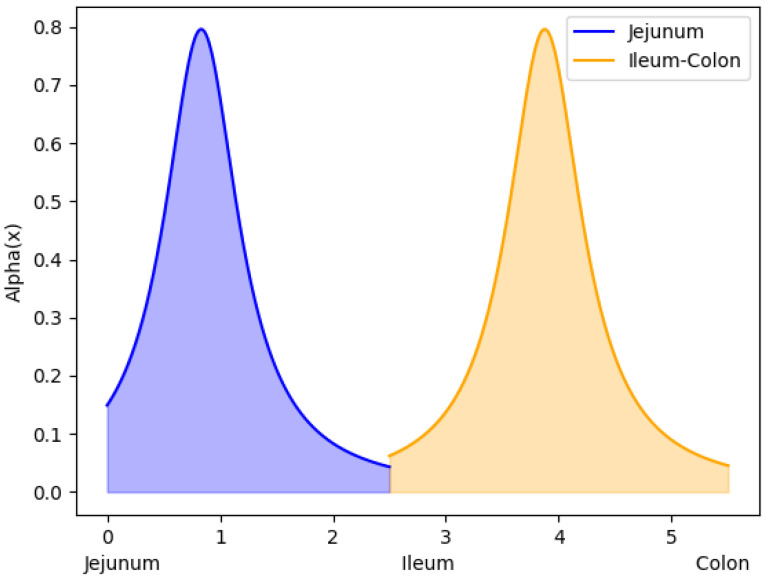
Example of alpha function distribution along the intestinal tract. It can be observed that the tail of the ileum–colon distribution falls on the colon, explicitly expressing the low absorption present in this segment.

$\lambda_{j}$ and $\lambda_{ic}$ are the model parameters, defining the form of $\alpha_{j}$ and $\alpha_{ic}$ distributions, as illustrated in Figure 3. Furthermore, the values of ${\tilde{L}}_{j}$ and ${\tilde{L}}_{ic}$ were set equal to $\frac{L_{j}}{3}$ and $L_{j}+\frac{L_{i}+L_{c}}{5}$ in order to achieve an appropriate positioning of the peaks of $\alpha_{j}$ and $\alpha_{ic}$ functions.

It is important to note that the $K_{j}$ and $K_{ic}$ parameters, which regulate drug passage from boluses to blood and vice versa, are inherently linked to intestinal permeability. Therefore, they can be physiologically linked to $\alpha_{j}$ and $\alpha_{ic}$, according to Equation (20) [44]:

(20) $$K_{l}=\frac{\alpha_{l}V_{bol}}{\pi r_{bol}^{2}\left(u+v_{b}\right)}\;\;\forall \;l=j,ic$$

The specific meaning and value of each parameter included in Equation (20) are described in Table 3. $V_{bol}$ denotes the volume of each bolus, which is equal to the volume of the duodenum prior to emptying. It is assumed that the boluses are spherical, with a radius indicated by $r_{bol}$.

On the whole, the model is defined by three parameters: $k_{d}$, $\lambda_{j}$, and $\lambda_{ic}$. These parameters can be determined, for every drug, through a least squares estimation, with the objective function ϕ described by Equation (21):

(21) $$\phi \left(\lambda_{j},\lambda_{ic},k_{d}\right)=\sum_{y=1}^{M}{\left(\frac{{\bar{C}}_{\mathrm{obs}}\left(t_{y}\right)}{\max_{y}\left({\bar{C}}_{\mathrm{obs}}\left(t_{y}\right)\right)}-\frac{{\bar{C}}_{\lambda_{j},\lambda_{ic},k_{d}}\left(t_{y}\right)}{\max_{y}\left({\bar{C}}_{\lambda_{j},\lambda_{ic},k_{d}}\left(t_{y}\right)\right)}\right)}^{2}$$

where $C_{obs}$ denotes the observed concentrations recorded from experimental data (Table 2), while *M* is the number of data points available in each specific dataset. The selection of normalization is guided by the necessity to streamline calculations, given that concentrations are expressed in disparate units across distinct studies. The simulations were conducted using Matlab^®^(v. R2023b, The Math Works Inc., Natick, MA, USA) software, with parameter estimation performed via the Matlab function *fmincon()*. In each simulation, the initial values of the parameters were varied in an attempt to identify a global minimum.

### 2.4. Model Validation

In order to validate the model, it was decided to test its ability to predict drug concentration in the peripheral blood compartment as a function of time for different dosages of two drugs (Aprepitant and Ketokenazole). Specifically, upon training the model with an initial dosage of 80 mg for Aprepitant and 600 mg for Ketokenazole, concentration curves were predicted for subsequent dosages of 125 mg for Aprepitant and 200 mg, 400 mg, and 800 mg for Ketokenazole. The results were compared with data consistent with those used for parameter estimation, derived from the same sources, for both Aprepitant [56] and Ketokenazole [62]. The remaining drugs were not included in the validation process due to the lack of consistent and homogeneous data sources.

Furthermore, additional validation was conducted by comparing the experimental effective intestinal permeability values with those provided by the model, which were calculated according to Equation (22):

(22) $$P_{eff}=\frac{r_{int}}{L_{j}}\int_{0}^{L_{j}}\alpha_{j}\left(x\right)\;dx+\frac{r_{int}}{L_{i}+L_{c}}\int_{L_{j}}^{L_{j}+L_{i}+L_{c}}\alpha_{ic}\left(x\right)\;dx$$

## 3. Results

### 3.1. Parameter Estimation Results

**Figure 4:**
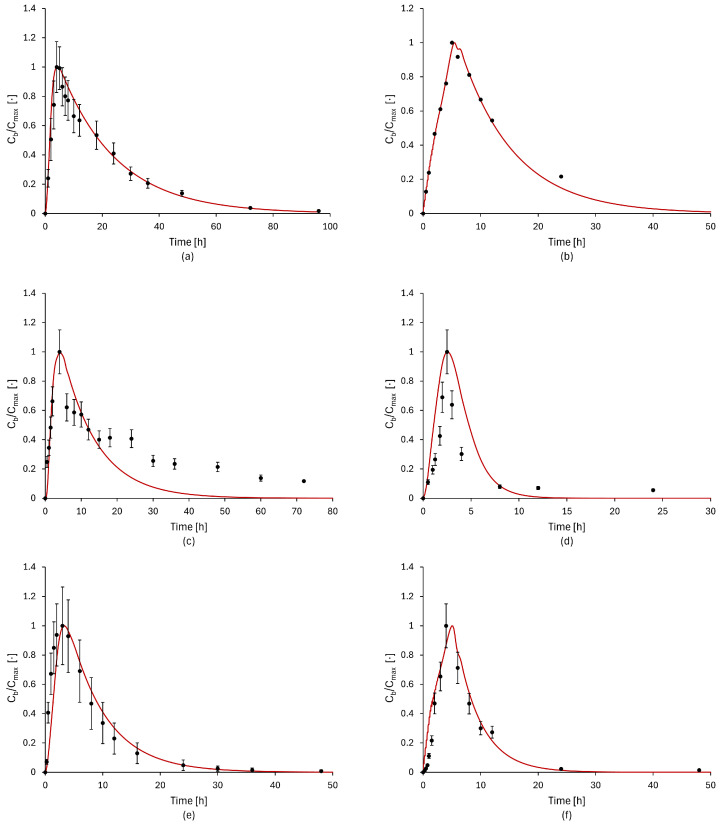

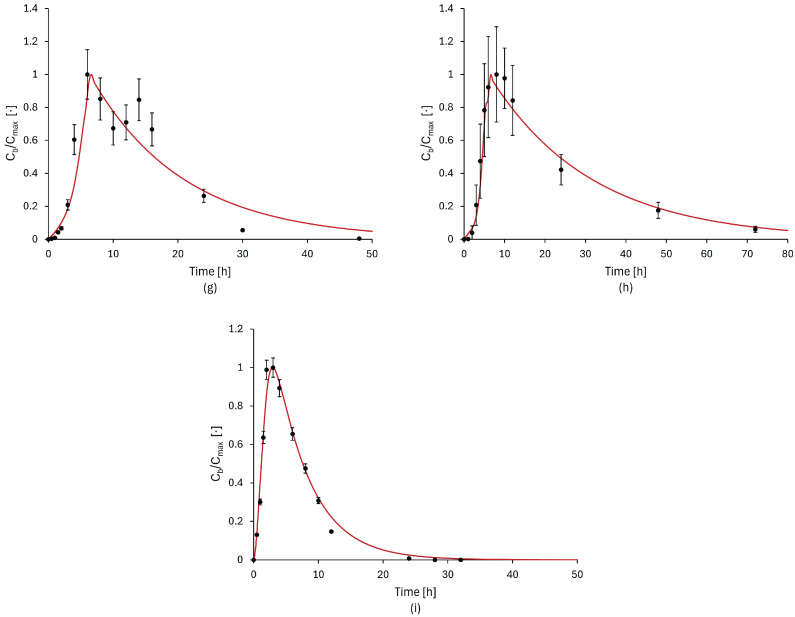
Comparison between model simulation results (red line) and experimental data (black dots) for nine different drugs: (**a**) Aprepitant (80 mg) [56], (**b**) Griseofulvin (125 mg) [57], (**c**) Etoricoxib [64], (**d**) Ketoprofen [63], (**e**) Linezolid [58], (**f**) Danazol [59], (**g**) Ibuprofen [61], (**h**) Fenofibrate [60], and (**i**) Ketokenazole (600 mg) [62]. Both the model and experimental concentrations were normalized with respect to their highest values.

The results of parameters estimation are reported in Table 4 and Figure 4.

In general, the concentration profiles obtained with the model are in good agreement with the experimental data. For the drug Aprepitant, the predicted curve may overestimate the concentration or reach the limit of the standard deviation after the peak. A similar, more pronounced issue is evident in the concentration profiles for the drugs Etoricoxib, Ibuprofen, and Ketoprofen. In these instances, the discrepancy in values may be attributed to noise in the experimental data or may be indicative of unusual circumstances during the gastric digestion process. In the case of the drug Ketokenazole, which was analyzed at four different initial dosages, the model demonstrated good adherence to the experimental data, with the exception of an error in anticipating the peak time in the concentration profile with an initial dosage of 800 mg. In the case of the remaining drugs, the parameter estimation results are satisfactory, with the model concentration profile fitting well to the experimental data, both in terms of concentration and peak time, as well as in the elimination/consumption phase.

### 3.2. Model Validation Results

**Figure 5:**
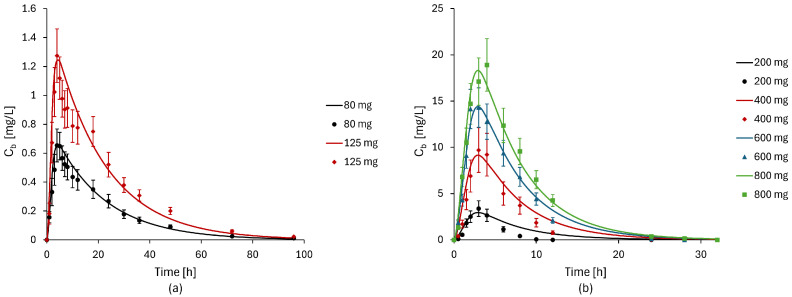
Results of the validation process for different dosages of Aprepitant [56] (**a**) and Ketokenazole [62] (**b**). The continuous lines indicate the predicted curves, while the individual points correspond to the experimental data.

The results of model validation are depicted in Figure 5.

Overall, the model predictions align well with experimental data. However, there are a few discrepancies observed for Ketokenazole. In particular, the 800 mg dosage prediction deviates in peak time and absolute value, while the curve for 200 mg overestimates concentration post-peak time.

**Figure 6:**
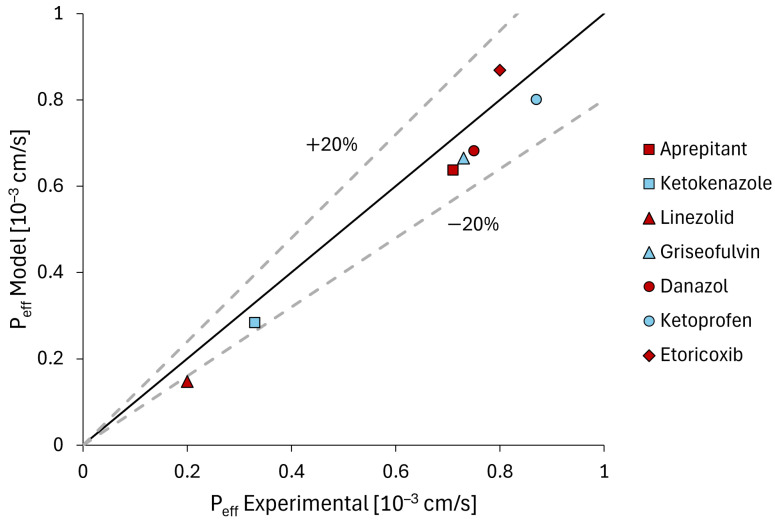
Comparison of experimental $P_{eff}$ values [53] with those obtained by model. To the best of our knowledge, the literature did not contain any information regarding the effective permeability of fenofibrate and ibuprofen. Consequently, these values were not included in the validation process.

With regard to the validation of effective intestinal permeability, Figure 6 illustrates a comparison between the $P_{eff}$ values derived from experimental data and those predicted by the model, in accordance with Equation (22). With the exception of Etericoxib, the observed underestimation of effective permeability for all drugs falls within a similar order of magnitude. In particular, the root mean square error (RMSE) was found to be approximately 0.064 · 10$10^{-3}$ cm/s, with a coefficient of determination ($r^{2}$) of 0.9935. Therefore, the values should be considered comparable despite the inherent approximations made, as this prevents an absolute correspondence.

## 4. Discussion

A number of approximations were made in the development of the model. Particularly, the predicted concentration profile is highly dependent on the concentrations present at the beginning of the digestive process, the definition of which is still imprecise. Furthermore, the influence of physical phenomena during the complex digestive process, such as the impact of food composition or lipids on drug molecules, was not considered. In addition, three physiological parameters are assumed to be constant: emptying frequency $f_{s}$, blood velocity in the intestine $v_{b}$, and bolus velocity *u*. Undoubtedly, a critical factor is the definition of $f_{s}$. Although this parameter is never constant, under standard conditions with a food and drug volume of approximately 1 L and regular digestion, its variation is minimal [65]. Nevertheless, it is acknowledged that the speed of digestion and absorption varies depending on the type of meal, specifically in terms of the concentration of proteins, carbohydrates, and lipids, as they have different digestion times [66]. This aspect could be partially incorporated into the model through a slight change in the dynamics of gastric emptying. In addition, the model can be expanded to incorporate upper GI drug absorption and the “first-pass effect”. This can be achieved by including additional compartments (such as the liver and wider peripheral circulations) where drug transfer and metabolism can markedly diminish bioavailability [67]. Ultimately, the Noyes–Whitney equation can be substituted with more complex relationships to account for incomplete drug dissolution.

In consideration of the aforementioned approximations and the limited number of parameters and simplicity of the equations describing absorption, the model predictions are a satisfactory result. The estimated curves demonstrated an adequate capacity for describing the concentration profile of the drugs considered in this study, particularly in the prediction of the time of maximum concentration. However, more substantial errors were observed in the concentration decay. This discrepancy may be attributed to the selection of the optimization algorithm, namely, ordinary least squares, which assigns greater weight to high values, which have a higher standard deviation. Consequently, the selected optimization method prioritizes the identification of the peak concentration time point. Clearly, the algorithm may undergo variations depending on the subsequent analyses to be performed.

Furthermore, a correlation between the absorption occurring in the ileum and colon and the magnitude of absorption can be observed. This is evident especially for the drugs ibuprofen and griseofulvin, where low values of $\lambda_{ic}$ and, thus, reduced effective permeability in the ileum and colon, imply a more pronounced decline in concentration. This aspect further supports the model’s robustness, demonstrating its ability to capture and explain the relationships between drug absorption and concentration dynamics.

A notable strength of this model is its minimal parameter count. In contrast, literature models that are widely employed in commercial software [68,69] incorporate numerous factors, such as the influence of food or micelles [70], resulting in a higher number of parameters. This can enhance the accuracy of the model, but it also requires an elevated computational cost, which necessitates the acquisition of a broader set of experimental data. However, there is often a paucity of quantitative data on chemical–physical processes occurring in specific human body compartments. The proposed model addresses this issue by describing a biological process with a minimal number of parameters while still achieving an acceptable level of accuracy. Therefore, the objective is to utilize this model during the initial stages of drug development, when a limited amount of experimental data is still accessible. This approach could facilitate an expeditious, preliminary assessment of drug absorption and bioavailability with minimal computational expense. Moreover, the model’s output could serve as an input for more sophisticated models, ultimately reducing their computational cost.

## 5. Conclusions

This research focused on the formulation and optimization of a mathematical model to simulate the activity of the gastrointestinal system during drug digestion and adsorption. The methodology adopted drew inspiration from two pre-existing models, which were merged and subjected to a series of adjustments in order to enhance their predictive accuracy, without excessively increasing the computational cost. Five anatomical compartments, including the stomach, duodenum, jejunum, ileum–colon, and peripheral blood circulation, were modeled as continued stirred tank reactors to depict the absorption kinetics for nine distinct drugs over time. The pivotal stage of the research entailed the optimization of the three model parameters, each endowed with a specific physiological significance. Validation of the model was conducted for two of the nine selected drugs, utilizing data from the literature covering a range of different initial dosages. In order to assess the robustness of the model, the effective intestinal permeability was also evaluated and compared to available data from the literature.

On the whole, the paper demonstrates, through a semiquantitative approach, the potential of a pharmacokinetic model with a limited number of parameters. Although validation was conducted on a modest number of drugs, the outcomes are encouraging. The results indicate that, despite the simplifications implemented, discrepancies with actual concentration profiles are minimal, predominantly associated with the phase of concentration decay. Consequently, in the context of drug development, this model may serve as a preliminary tool for drug absorption and bioavailability evaluation, employing limited experimental data without a significant computational cost. However, as a future direction, a larger number of drugs will be considered to facilitate a more comprehensive validation process.

## Figures and Tables

**Table 1:** Drug dissolution parameters. The values of $S_{0}$ were obtained from [52] for each drug.

Drug	$S_{0}$ (mol/L)	*D* ($10^{-9}$ $m^{2}$/s)	ρ (g/mL)	${\mathrm{pk}}_{a}$	Reference
Aprepitant	$1.94\cdot 10^{-2}$	0.63	1.51	9.15	[53]
Griseofulvin	$4.25\cdot 10^{-5}$	0.7	1.38	17.7	[53]
Linezolid	$4.3\cdot 10^{-3}$	0.67	1.12	1.8	[52]
Danazol	$1.5\cdot 10^{-6}$	0.68	1.21	4.7	[53]
Fenofibrate	$6.9252\cdot 10^{-7}$	0.66	1.18	4.7	[53]
Ibuprofen	$1\cdot 10^{-4}$	0.61	1.6	5.3	[52]
Ketokenazole	$1.225\cdot 10^{-5}$	0.66	1.38	6.5	[53]
Ketoprofen	$2\cdot 10^{-4}$	0.7	1.6	4.45	[52]
Etoricoxib	$0.6\cdot 10^{-4}$	0.59	1.41	4.96	[52]

**Table 2:** Sources of experimental data considered for each drug.

Drug	Reference
Aprepitant	[56]
Griseofulvin	[57]
Linezolid	[58]
Danazol	[59]
Fenofibrate	[60]
Ibuprofen	[61]
Ketokenazole	[62]
Ketoprofen	[63]
Etoricoxib	[64]

**Table 3:** Physiological parameters used in jejunum and ileum–colon models.

Symbol	Parameter	Value	Reference
δ	Peripheral circulation duration	90 s	[47]
$f_{e}$	Emptying frequency	$1.66\cdot 10^{-3}$ Hz	[44]
$v_{b}$	Blood velocity in the intestine	0.21 m/s	[44]
*u*	Average bolus velocity in the intestine	1 m/h	[44]
$Q_{b}$	Blood flow rate in the intestine	0.033 L/s	[47]
$L_{j}$	Length of the jejunum	2 m	[47]
$L_{i}$	Length of the ileum	4 m	[47]
$L_{c}$	Length of the colon	1.5 m	[47]
$V_{b}$	Blood volume in peripheral circulation	4 L	[44]
$r_{int}$	Intestine radius	0.01 m	[47]

**Table 4:** Values of estimated parameters.

Drug	$\lambda_{j}$ [$10^{-3}\cdot$ $m^{2}$/s]	$\lambda_{\mathrm{ic}}$ [$10^{-3}\cdot$ $m^{2}$/s]	$k_{d}$ [$10^{-5}\cdot$ $s^{-1}$]
Aprepitant	5.157	5.100	2.012
Ketokenazole	3.506	1.311	8.095
Griseofulvin	5.858	4.949	4.695
Linezolid	1.184	1.195	5.352
Irbesartan	6.860	5.994 $\cdot 10^{-3}$	5.036
Danazol	7.325	4.029	10.287
Fenofibrate	104.031	73.810	0.836
Ibuprofen	286.936	18.827	3.074
Ketoprofen	7.000	6.000	25.343
Etoricoxib	6.993	6.988	2.011

## Data Availability

The original contributions presented in the study are included in the article; further inquiries can be directed to the corresponding author.
